# The mechanism for the specificity of gaze direction: Inhibiting background location

**DOI:** 10.1177/20416695241270303

**Published:** 2024-08-12

**Authors:** Airui Chen, Weixia Han, Wei Wang, Bo Dong

**Affiliations:** Department of Psychology, 66339 Suzhou University of Science and Technology, Suzhou, China

**Keywords:** stroop effect, arrow direction, gaze direction, background location

## Abstract

The experiment combined the spatial Stroop paradigm to examine the effect of background location on the perception of arrow or gaze direction in the vertical dimension by manipulating the congruence between the target direction and background location, and to validate a possible cognitive mechanism for gaze direction specificity – inhibiting background location. The results showed that when subjects were required to identify the target direction in a Stroop task (Experiment 1), the gaze cue failed to induce the Stroop effect. However, when subjects were required to judge the congruence between the target direction and the background location (Experiment 2), the gaze cue and the arrow cue both induced the Stroop effect. This suggests that “ inhibiting background location” is responsible for the elimination of the spatial Stroop effect by gaze direction, which may one of the mechanisms for gaze direction specificity.

Humans change their focus of attention based on external stimuli. For example, drivers observe road conditions through traffic signs (arrow direction), and athletes choose the direction of attack according to the gaze of their teammates (gaze direction). Although both arrow and gaze directions can direct our attention (Yokoyama et al., 2020), a number of studies have found that social attention (gaze) is special and that our brain processes gaze with specialized mechanisms (Jones et al., 2010; Liu et al., 2021; Shepherd et al., 2009; Teufel et al., 2010). Specificity refers to how directional cues that are social in nature differ from other directional cues. Behavioral studies have shown that, compared with arrows, the attentional effects of gaze are not less affected by top-down factors, such as cue predictivity and the color congruency of cues and targets (Friesen et al., 2004; Ristic et al., 2007). Additionally, items or objects in the direction of other's gaze are better remembered compared to arrows (Dodd et al., 2012; Gregory & Jackson, 2017). ERP studies have found that the brain's processing of gaze direction is already significantly different from that of arrows when the stimulus appears at approximately 200 ms (Marotta et al., 2019; Schuller & Rossion, 2001). fMRI and brain injury studies have found that activation of the right half of the brain's superior temporal sulcus (STS) activation is only associated with the processing of gaze direction (Akiyama et al., 2006; Shepherd, 2010). Accordingly, researchers believe that there is a module in the human brain dedicated to detecting and processing gaze direction, i.e., the gaze direction detector (Baron-Cohen, 1995; Ji et al., 2018). Therefore, although existing research has basically clarified the behavioral performance and the corresponding physiological structure of gaze direction with respect to specificity, the mechanistic question of how our brain realizes the specificity of social gaze (i.e., cognitive mechanism of gaze direction specificity) has not been satisfactorily answered.

Inhibiting background location may be one mechanism of specificity in the mental processing of gaze direction. As directing stimuli, arrows, and gaze mainly point to a direction or location in space, i.e., the target direction, such as an arrow pointing to the right. Also, the arrows and gaze stimuli have the property of physical location, i.e., their background location, such as the arrow above presented on the left of the screen. In terms of stimulus composition, the target direction and their background location convey independent information. However, this does not mean that the corresponding processes of these two kinds of information in our brain are independent of each other. For example, participants responded more quickly to left-pointing arrows (target direction) presented on the left side of a screen (background location) than to left-pointing arrows on the right (Clark & Brownell, 1975; Shimamura, 1987). This suggests that when our brain extracts the pointing direction of an arrow, it indeed cannot ignore the background location. According to the dimensional overlap model, the more overlap in the cognitive processing of task-related stimuli (e.g., target direction), response mode (e.g., the arrangement of response keys or the left and right hands), and unrelated stimuli (e.g., background location), the more the background information interferes with or facilitates the task (Kornblum et al., 1990). It is worth noting that it is unclear whether the perception of gaze direction is independent of the perception of their background location. If we can mentally inhibit the background location during the processing of gaze direction, we can exact the direction of gaze more quickly, stably and accurately, which is consistent with the view of the specificity of social gaze.

The spatial Stroop task is an effective means of validating the “inhibit background location” mechanism. The Stroop task investigates the ability to inhibit unrelated information during target processing. In the task, researchers present arrows pointing to a direction on the screen to produce a conflict between the target direction and their background location. For instance, present a downward pointing arrow at the top of the screen. Participants only needed to judge the indicated direction of the arrow and inhibit its physical location (i.e., background location). Researchers have observed a spatial Stroop effect, i.e., participants have shorter RTs under congruent conditions than under incongruent conditions. That is, a spatial Stroop effect occurs, indicating that individuals processing the arrow direction (target direction) cannot inhibit the arrow location (background location) (Funes et al., 2007; Lupiáñez & Jesús Funes, 2005). This logic can be used to answer the question of whether we can mentally inhibit the background location of stimuli during the perception of gaze direction. According to this logic, if the congruency of gaze's indicated direction and its background location can also induce the spatial Stroop effect, we cannot inhibit the background location of stimuli while extracting the direction indicated by the gaze; otherwise, if the gaze direction fails to induce a spatial Stroop effect, it suggests that gaze direction can inhibit background location and it has a high degree of specificity. Some researchers have used the spatial Stroop paradigm to validate the specificity of gaze (Chen et al., 2022; Hemmerich et al., 2022; Marotta et al., 2018; Román-Caballero et al., 2021a, 2021b; Tanaka et al., 2023). Note that, except for the location and background of the target, there also exists a response location in the Stroop task, i.e., the response hands or the spatial arrangement of the response keys. To exclude interference of the response location, we set the target and their background location in the vertical dimension (up and down) and set the response location in the horizontal dimension (left and right buttons) in this study. More importantly, a single eye is an ellipse with a long horizontal axis, and thus, the directivity of the left and right gaze is quite robust, which causes a larger gaze cueing effect, and the up and down gaze is weaker and produces a less attentional effect (Alais et al., 2018; Lee et al., 2019). With large gaze cue effects, even if the gaze cue effects are found to be independent of background location, the possibility of “large gaze cue effects masking small background location effects” cannot be ruled out. However, if the cue effect is smaller and still unaffected by background location, we can better infer that our brain indeed inhibits background location when judging gaze direction. Thus, we can better provide evidence for the mechanism underlying inhibiting background location.

Based on the spatial Stroop task, we manipulated the congruency of target direction and background location and designed 2 (target type: arrow, gaze) × 2 (congruency of target direction and their background location: congruent, incongruent) experiments to investigate the influence of background location on the perception of gaze and arrow direction in the vertical axis and to verify the possible mechanism underlying inhibiting the background location. In experiment 1, the participants were asked to judge only the indicated direction of two types of targets, i.e., arrows and gaze, inhibiting their background location. This allowed the inference of background location on gaze and arrow perception to be examined in a natural state. If our brain achieves gaze perception through the mechanism underlying inhibiting background location, the congruency of gaze direction and background location will not influence the gaze perception, and the modulation effect on the perception of arrows rather than gaze will be observed. To further verify that the results of Experiment 1 were due to inhibiting background location, Experiment 2 actively introduced background location information into the task and asked the subjects to judge the consistency between the direction indicated by the target (i.e., the target direction) and the background location of the target (i.e., the background location). At this point, subjects needed to accurately perceive not only the arrow or gaze direction, but also correctly process the background location. If the specificity of the gaze direction in Experiment 1 was caused by inhibiting the background, the role of the specificity would be lost in Experiment 2. In other words, the gaze exhibits the same Stroop effect as the arrows.

## Experiment 1

**Figure 1.**
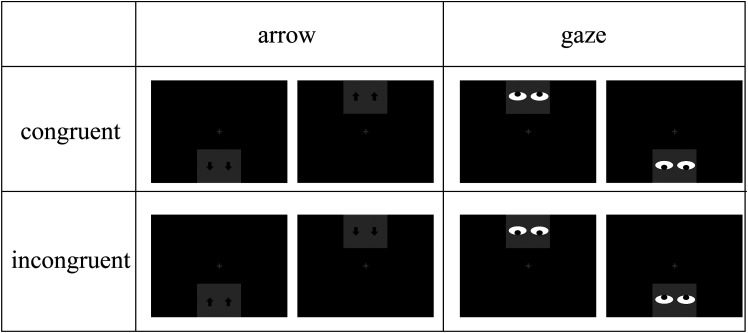
The four conditions in this study: arrow-congruent, arrow-incongruent, gaze-congruent, and gaze-incongruent.

This experiment was a 2 (target type: arrow, gaze) × 2 (congruency of target direction and their background location: congruent, incongruent) within-subjects design. We aimed to investigate whether the perceptual judgments of gaze and arrow directions were influenced by the background locations of the targets in the vertical direction, which can help to verify the possible cognitive mechanism underlining the specificity of social gaze. The stimuli are shown in Figure 1. In the congruent trials, the direction of gaze or arrow was consistent with their background location. For example, a downward pointing arrow and a downward looking gaze were presented at the bottom of the screen. In the incongruent trials, the direction of gaze or arrow was opposite to their background location. For example, an upward pointing arrow and an upward looking gaze were presented at the bottom of the screen. We examined the participants’ perception of the targets and calculated the reaction time and accuracy in the target-direction discrimination task. In the experiment, subjects were only required to make judgements on the direction of gaze or arrow cues (i.e., target direction) without paying attention to the location of the arrow or gaze cues (i.e., background location), in order to verify whether the background location information interfered with the judgement of target direction in the natural state. If we extract gaze direction by inhibiting background information to enable specific processing of gaze, the congruency of target direction and their background location would not interfere with the perception of gaze direction. It is reasonable to infer that the Stroop effect would be found for arrow and not gaze. In contrast, if congruency influences the perception of gaze direction, the Stroop effect could be observed in both gaze and arrow conditions.

### Method

#### Participants

Our effect of interest was the interaction of target type and the congruency of target direction and their background location. The sample size was determined based on previous relevant studies (Marotta et al., 2018; Xu et al., 2011), and a two-tailed power analysis using G*Power (Vision 3.1.9) confirmed that a sample size of 15 participants would afford 80% power to detect an attentional effect induced by social or nonsocial cues. We chose a medium *f* value and conducted an a priori power analysis, with f = 0.27, α = 0.05, 1−β = 0.8, number of groups = 1, number of measurements = 4, correlation among repeated measures = 0.5, and nonsphericity correction ε = 1. The sample size for this effect was calculated to be 21 participants.

A total of 22 participants (17 females and 5 males; M_age__ _= 20 years, SD_age__ _= 1) were recruited for the experiment. They all had a normal or corrected-to-normal vision. All the participants were naive to the purpose of the research and were rewarded ¥ 5 after the experiment. One participant was excluded, with an overall accuracy lower than 90%. The actual sample size was 21, which met the a priori sample analysis. Before the experiment, all the participants were given informed consent.

#### Apparatus and Stimuli

The experiment was programmed with E-prime 1.0 and run on a Lenovo (M690E) microcomputer with a discrete graphics card. A 17-inch Lenovo monitor (9227-AE6) with 1024 × 768 (px) resolution and a refresh rate of 60 Hz was used. The experimental scenes and parameters were adopted from Marotta and his colleagues (2018) and are shown in Figure 1. All the stimuli were presented on a black background (RGB: 0, 0, 0; luminance: 0.21 cd/m^2^). The fixation was a white cross (RGB: 255, 255, 255); luminance: 59.04 cd/m^2^, with a visual angle of 1° × 1°. For the gaze, we present a human face in a gray square (RGB: 128 128 128; luminance: 33.86 cd/m^2^; 7.1° × 5.4°) below or above the fixation, with an eccentricity of 9° visual angle. The arrow and gaze are presented in the center of the gray square. The single left or right arrow was a 1.1°×1.4° visual angle. The left or right eye had a 2.9° × 1.4° visual angle. The two arrows or eyes were separated by a 2.9° visual angle.

#### Procedure

The participants sat 60 cm from the screen in a dark room, with their heads stabilized on the chin rest. In each trial, after fixation onset for 1000 ms, the arrow or the gaze was randomly presented below or above the fixation. The participants should judge the direction of the target, pressing the “Z” key for the up direction and the “M” key for the down direction as soon as possible. If they made an incorrect response or did not respond in the required period (1500 ms), feedback noise (220 Hz) was presented for a duration of 500 ms. The experiment included four conditions (target type × congruency of target direction and their background location), i.e., gaze-congruent, gaze-incongruent, arrow-congruent, and arrow-incongruent, with 64 trials for each condition. All 256 trials were randomly separated into 4 blocks. The participants could rest for 2 min after finishing each block. Before the formal experiments, the participants finished a practice block with 32 trials to become familiar with the task.

#### Results

The accuracy of the 21 participants was 94.92%∼100% (*M *= 98.19%, *SD *= 2.05). Table 1 shows the means and SEs of RT and ACC for each condition.

**Table 1.** Mean and standard error (SE, in brackets) of reaction time and accuracy for each condition.

	Congruent		Incongruent		
Target type	RT	ACC	RT	ACC	Stroop
Arrow	662.37 (21.68)	98.21 (0.47)	686.31 (23.31)	98.36 (0.35)	23.94*
Gaze	703.21 (23.09)	97.99 (0.50)	693.13 (23.59)	98.21 (0.47)	−10.08

Note: Stroop = RT _Incongruent_ -RT _congruent_; *p < .05, **p < .01.

**Figure 2.**
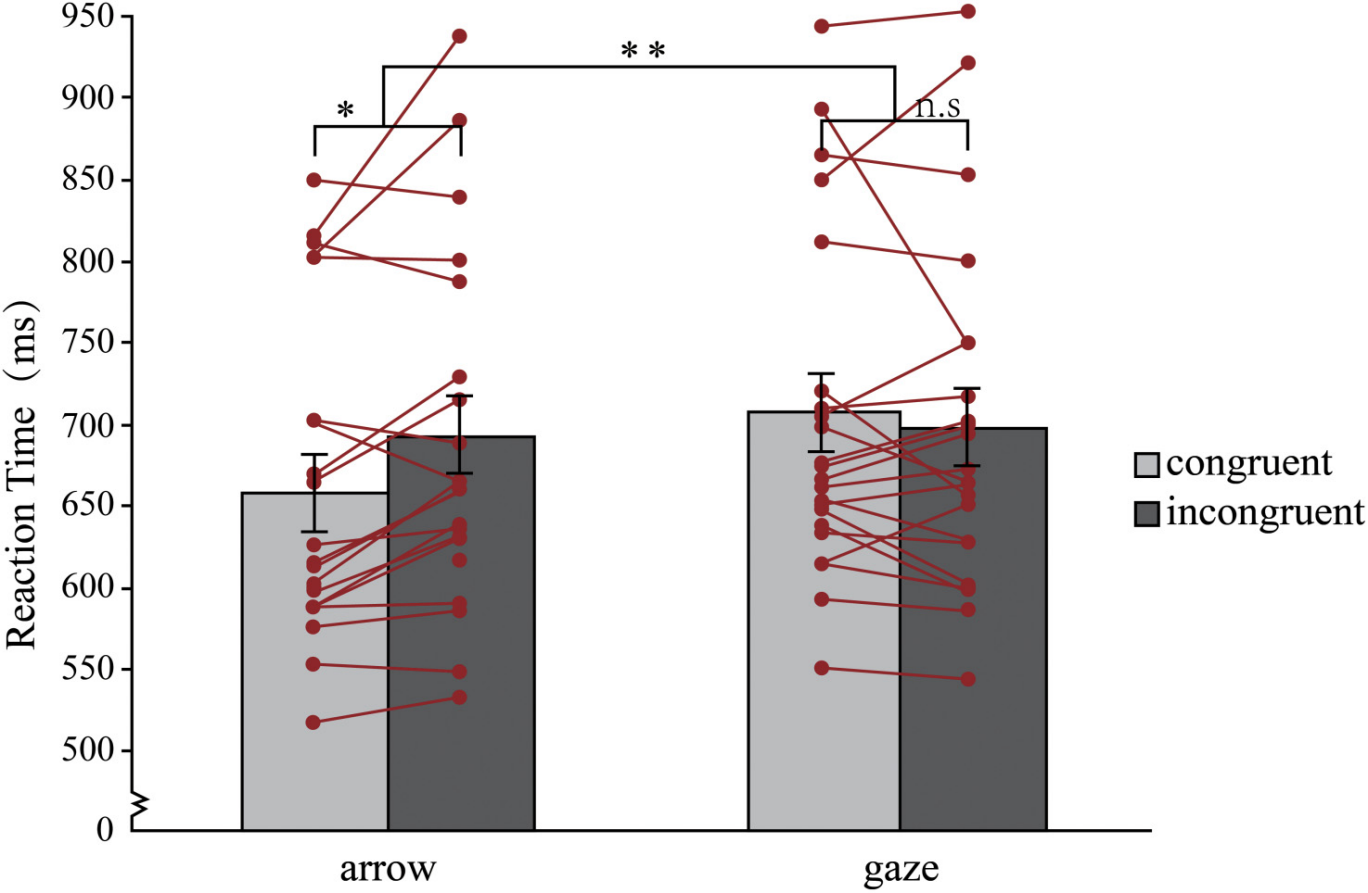
Mean RTs for each condition in experiment 1. Error bars represent standard errors. The lines and points represent the mean RT_congruent_ and RT_incongruent_ for each participant. *p < .05, **p < .01, n.s. = not significant.

A two-way RM-ANOVA with target type (arrow, gaze) and congruency of target direction and their background location (congruent, incongruent) on mean RTs revealed a significant main effect of target type, *F* (1, 20) = 18.74, *p *< .001, *η_p_*^2^^ ^*= *0.48, 95% CI = [−35.31, −12.35], 1−*β* = 1.00, and the target type*congruency interaction, *F*(1, 20) = 15.16, *p *= .001, *η_p_*^2^^ ^= 0.43, 1−*β* = 1.00. No significant effect was observed for the main effect of congruency, *F*(1, 20) = 0.76, *p *= 0.39, *η_p_*^2^^ ^= 0.037, 95% CI = [−23.52, 9.64], 1−*β* = 0.06. As illustrated in Figure 2, two paired t tests revealed no significant differences between the congruent and incongruent conditions when the gaze was presented, *t*(20) = 1.09, *p *= .29, *d *= 0.24, 95% CI = [−29.38,9.23], 1−*β* = 0.18. This finding suggests that the gaze did not produce the classic Stroop effect (*M ± SE = *−10 ms ± 9 ms). However, when an arrow was presented, significant differences were found between the congruent and incongruent conditions, *t*(20) = 2.70, *p *= .014, *d *= 0.59, 95% CI = [−42.48, −5.42], 1−*β* = 0.73, which showed the Stroop effect (*M ± SE = *24 ms ± 9 ms).

### Discussion

In Experiment 1, with the spatial Stroop task, the participants judged only the direction of the gaze or arrow, inhibiting the background location of the targets. This was designed to test whether background location interferes with the perception of target direction in a more natural state. The findings show that gaze is essentially different from arrows in the spatial Stroop effect, with arrows producing classic Stroop effects and gaze not producing such effects. This suggests that the congruency of gaze direction (relevant/semantic information) and their background location (irrelevant information) cannot interfere with the speed at which gaze direction is perceived. We hypothesized that our brain may achieve the specificity of gazes through a mechanism that effortlessly inhibits the irrelevant background information surrounding the gaze. To further test this hypothesis, in experiment 2, we designed a task in which the participants were asked to respond after processing the background information. In experiment 2, the spatial Stroop task was still adopted; simultaneously, the spatial background information was included in the task. The participants were asked to judge whether the direction indicated by the target (i.e., the target direction) was consistent with the location of the target (i.e., the background information). In this experiment, the participants not only needed to accurately perceive the arrow or gaze direction but also needed to correctly process the location of the targets. If the specificity of the gaze direction is due to inhibiting the background information, the gaze should produce the same spatial Stroop effect as the arrow.

## Experiment 2

### Method

#### Participants

The priori power analysis and the planned sample size were the same as those of experiment 1. Twenty-four participants (14 females and 10 males; *M_age__ _* = 20 years, *SD_age__ _* = 1) were recruited for the experiment. They all had normal or corrected-to-normal vision. All the participants were naive to the purpose of the research and were rewarded ¥ 5 after the experiment. Three participants were excluded, with an overall accuracy lower than 90%. The actual sample size was 21, which met the a priori sample analysis. Before the experiment, all the participants were provided informed consent.

#### Apparatus, Stimuli, and Procedure

The apparatus, stimuli, and procedure were the same as those in Experiment 1, except for the task instructions. If the target direction was the same as their background location, the participants were asked to press the “Z” key. If the target direction was opposite to their background location, the participants pressed the “M” key.

### Results

The accuracy of the 21 participants was 91.5%∼97.88% (*M *= 96.5% *SD *= 1.08). Table 2 shows the means and SEs of RT and ACC of each condition.

**Table 2.** Mean and standard error (SE, in brackets) of reaction time and accuracy for each condition.

	Congruent		Incongruent		
Target type	RT	ACC	RT	ACC	Stroop
Arrow	952.25 (21.09)	95.33 (1.16)	1049.52 (32.46)	96.83 (0.58)	97.27***
Gaze	1015.92 (31.81)	96.93 (0.39)	1140.86 (40.58)	96.95 (0.32)	124.94***

Note: Stroop = RT _Incongruent_ -RT _congruent_; *p < .05; **p < .01.

**Figure 3.**
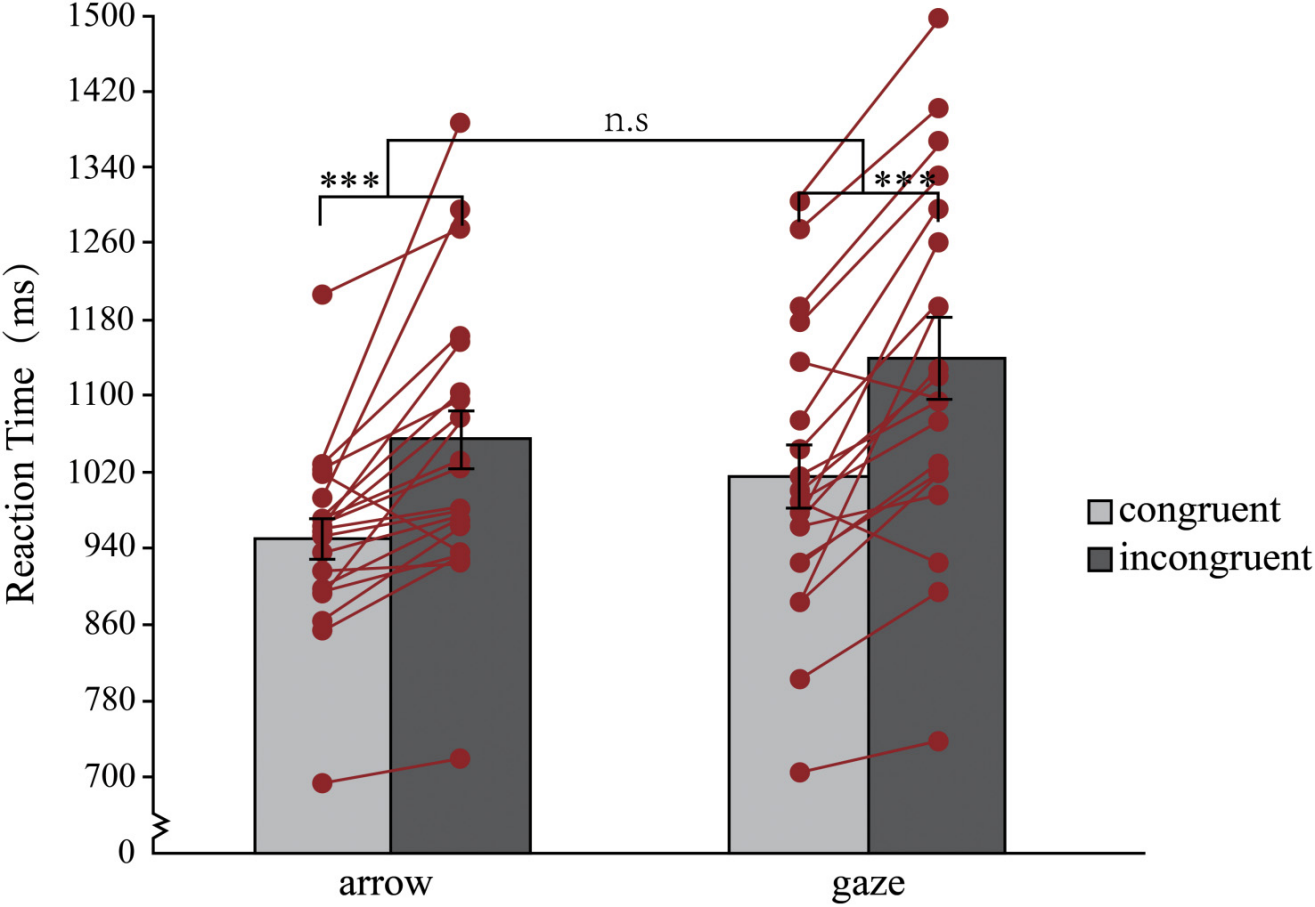
Mean RTs for each condition in experiment 2. Error bars represent standard errors. The lines and points represent the mean RT_congruent_ and RT_incongruent_ for each participant. *p < .05, **p < .01, n.s. = not significant.

A two-way RM-ANOVA with target type (arrow, gaze) and congruency of target direction and their background location (congruent, incongruent) on mean RTs revealed a significant main effect of target type, *F* (1, 20) = 13.95, *p *= .001, *η_p_*^2^^ ^= 0.41, 95% CI = [−120.79, 34.22], 1−*β* = 1.00, and congruency, *F*(1, 20) = 44.33, *p *< .001, *η_p_*^2^^ ^= 0.69, 95% CI = [−145.91, 76.30], 1−*β* = 1.00. No significant effect was observed for the target type × congruency interaction, *F*(1, 20) = 1.17, *p *= .29, *η_p_*^2^^ ^= 0.055, 1−*β* = 0.080 (see Figure 3).

To further determine whether the gaze and arrows produced the Stroop effect, we calculated the Stroop effect (RT _Incongruent_ −RT _congruent_) for each participant and then conducted an on-sample t test. There was a significant Stroop effect for both the gaze and arrows. When arrows were presented, a significant Stroop effect (97 ms ±21 ms) was observed, *t*(20) = 4.55, *p *< .001, *d *= 0.99, 95% CI = [−141.87, 52.68], 1−*β* = 0.99. Additionally, when gaze was presented, a significant Stroop effect (125 ms ± 21 ms) was observed, *t*(20) = 6.05, *p *< .001, *d *= 1.32, 95% CI = [−168.04, −81.82], 1−*β* = 1.00. Then, we conducted a paired t test to compare Stroop effect size between arrows and gaze and found no significant differences *t*(20) = 1.08, *p *= 0.29, *d *= 0.23, 95% CI = [−25.71,81.02], 1−*β* = 0.17. The findings above suggest that gaze and arrows produce the same Stroop effect.

## General Discussion

Based on the spatial Stroop task, this study investigated the effects of background location on the perception of gaze and arrow direction (i.e., target direction) in the vertical direction by manipulating the congruency of target direction and their background location. Particularly, we designed two tasks that required inhibiting the background information (experiment 1) or processing the background information (experiment 2) to examine how we perceive gaze to differ from other directing stimuli, such as arrows, and whether inhibiting background information was the possible mechanism of gaze direction specificity. First, consistent with previous findings, we demonstrated that an obvious Stroop effect was shown for arrows but not for gaze when the participants were asked to judge only the target direction, inhibiting background location (experiment 1). This suggests that our brain may effortlessly inhibit their background location of gaze and extract the direction of the gaze to achieve the specific processing of gaze. Second, we revealed the same Stroop effects for gaze and arrows when the task demanded involved background information, i.e., the participants were asked to judge the consistency of target direction and their background location, which further suggests that inhibiting background location is the possible reason why the two types of stimuli showed different effects in experiment 1. Overall, our results indicate the possible mechanism of social gaze specificity (i.e., inhibiting background location).

Consistent with previous findings, our study found that arrows can induce the Stroop effect. Marotta and his colleagues (2018) observed a 23 ms Stroop effect for arrows in the horizontal direction, which is very similar to the results of Experiment 1 (Stroop effect: 24 ms). This showed that the spatial Stroop effect induced by arrows is quite stable, thus suggesting that the perception of arrow location interferes with the perception of arrow direction. In other words, our brain cannot inhibit background location to extract the direction of arrows. According to this view, if we strengthen the saliency degree of arrows, the arrow-induced spatial Stroop effect is further enhanced. For example, Pires et al. (2017) found that against a white background, an arrow with a 7.15° visual angle (approximately 5 times the length of the arrow in our study) produced a 60 ms∼90 ms Stroop effect (Pires et al., 2017). Even without the Stroop task, studies have also found that participants cannot inhibit background information when they process arrows. For instance, Fan and his colleagues (2018) found that the distracters surrounding an arrow can interfere with the processing of the arrow (Fan et al., 2018). Conversely, when studies presented mosaics to reduce the saliency degree of background location, the spatial Stroop effect was obviously undermined and even disappeared (Román-Caballero et al., 2021a, 2021b). This further indicates that we indeed cannot inhibit or suppress background location in the process of perceiving arrows.

Unlike arrows, the task of judging gaze direction doesn't show a classic Stroop effect. Previous empirical research and theoretical explanations have sought to elucidate the specificity of gaze within the Stroop paradigm (Chen et al., 2022; Marotta et al., 2018; Narganes-Pineda et al., 2023; Román-Caballero et al., 2021a, 2021b; Tanaka et al., 2023; Hemmerich et al., 2022). Building upon these efforts, we specifically suggest that “inhibiting background location” is the mechanism through which gaze specificity manifests. In Table 3, we outline the findings from both our study and earlier research. When subjects process either gaze or arrow stimuli, they deal with direction/location information across several aspects: Pointing Direction (a); Spatial Location (b); Task-related (e). The Stroop paradigm involved manipulation of the target's direction (a) and its spatial location (b). Participants must also focus on the center of the screen during keypress tasks, making their responses according to the spatial positions of the keys (e). Taking into account all three processes and following the principle of simplicity, we can derive the following outcomes in Table 3. In the case of the arrow, under congruent conditions, processes of a, b, and e are all active. The congruency between b and e results in the Simon effect, and the congruency between b and a leads to the Stroop effect. Both effects contribute to quicker responses in congruent situations than in incongruent ones. In studies on gaze perception by Chen et al. (2022), Marotta et al. (2018), Hemmerich et al. (2022), Román-Caballero et al. (2021a, 2021b), and Tanaka et al. (2023), the suppression of process b arises from multiple causes. For the gaze cue, due to various reasons (such as the insignificance of directional information, the demanding nature of the task, the inhibition of all irrelevant information due to numerous peripheral stimuli, or the need for more attentional resources for processing because of the social nature of gaze), the b (i.e., spatial location) is suppressed when the cue is presented (before the button is pressed). Consequently, the Simon effect between ‘b’ and ‘e’ undergoes a strong reversal due to the negative priming effect. It's crucial to recognize that there could be a conflict between processes b and a, yet since neither involves the response or negative priming directly, the outcome of their interaction remains ambiguous. They could lead to a diminished conflict effect or potentially neutralize each other, resulting in no discernible effect. Therefore, the reversed Stroop effect occur. Specifically, we acknowledge the concept of inhibition as detailed in the dual-stage account (Tanaka et al., 2023). However, we believe this inhibition isn't tied to processing time windows, since Roman-Caballero et al. (2021b) did not observe a Stroop effect in gaze under asynchronous conditions. At an algorithmic level, we suggest the presence of process b's inhibition. This approach addresses the theoretical gaps previously highlighted and supports the computational modeling of gaze attention dynamics. Crucially, our theories regarding processes a, b, and e can be empirically tested. For example, if the response task's direction (e) is unrelated to the stimulus direction, then processes b and e do not interact, removing the conflict between b and e and thus transforming the reversed Stroop effect (e.g., Marotta et al., 2018) into a non-Stroop effect (as seen in Experiment 1 of our study). When the task directly engages process b, b is actively engaged rather than inhibited, which results in the traditional Stroop effect (as observed in Experiment 2 of our study). Following this logic, if the task lacks a directional component (such as color judgment, as proposed by Narganes-Pineda et al., 2023), then processes a, b, and c remain inactive, leading to the absence of a Stroop effect. Furthermore, our interpretation suggests that presenting peripheral stimuli earlier indicates that there is no need to inhibit the background location when the cue appears. Consequently, the conflicts between b and e, as well as between b and a, are resolved, thereby eliminating the Stroop effect.

**Table 3.** Comparing studies on the gaze-stroop paradigm within the framework of “inhibiting background location.

		Chen et al., 2022; Hemmerich et al., 2022; Marotta et al., 2018; Román-Caballero et al., 2021a, 2021b; Tanaka et al., 2023	Exp.1 in our study	Exp.2 in our study	Narganes-Pineda et al., 2023
Pointing Direction (a)		Left or right	Up or down	Up or down	Left or right
Spatial location (b)		Left or right	Up or down	Up or down	Left or right
Task (e)		Discriminating pointing direction (Left or right)	Discriminating pointing direction (Left or right)	Discriminating the consistency of cue direction and position	Color-discrimination
Arrow-congruent condition	process	a, b, e activated; mutually beneficial; b + e (Simon); b + a (Stroop);	a, b, e activated; b, e do not influence each other; b + a (Stroop);	a, b, e activated; b, e do not influence each other; b + a (Stroop);	a, b, e not activated and do not influence each other
	Result	Congruent effect	Congruent effect	Congruent effect	No effect
Gaze-congruent condition	process	a, e activated; b inhibited; e-b (reversed Simon); a-b (uncertain);	a, e activated; b inhibited; b, e do not influence each other; a-b (uncertain);	a, b, e activated; b, e do not influence each other); b + a (Stroop);	a, b, e not activated and do not influence each other
	Result	Reversed congruent effect	No effect	Congruent effect	No effect

It is worth noting that apart from background-location information according to these studies utilizing the Stroop paradigm, processing other types of background information struggles to impact gaze direction perception. Xu et al. (2011) adopted the rapid serial visual presentation (RSVP) paradigm and found that the cueing effect of arrows disappeared, but the gaze cueing effect persisted, which suggests that the perception of gaze direction is not influenced by such background information, i.e., perceptional load. Even when gaze is neither informative nor task-relevant, researchers still observe an attentional effect of gazes (Frischen et al., 2007; Hietanen et al., 2008; Wang et al., 2020). Recent studies have revealed that in the early stages of processing (P1 and N1), there is no significant difference in the processing of arrow and gaze, but in the later stages of processing (N2 and P3), there are significant differences between arrows and gaze (Román-Caballero et al., 2021b). This suggests that in the later stage of processing, there may be an additional component (FFA, related to face processing) that eliminates the conflict caused by the background location of faces, which leads to inhibiting background information in the perception of gaze. In addition, from the physiological view, the STS in the temporal lobe processes gaze direction, while the fusiform gyrus in the occipital lobe is responsible for face processing. These belong to different regions (Kanwisher et al., 1997; McCarthy et al., 1997; Nummenmaa et al., 2010; Pitcher & Ungerleider, 2021). The STS contains clusters of neurons encoding gaze direction, and its activity is independent of head direction and the physical features of the face (Pfeiffer et al., 2013). In other words, the brain has a hardware basis for the mechanism “inhibiting background location.”

The cognitive mechanism that inhibits background information during gaze processing is significant for evolution, cognition, social interactions, and autism therapy. Evolutionarily, this process enables humans to concentrate on the direction of others’ gazes, facilitating quicker identification of potential threats or intentions, thereby enhancing survival chances. Cognitively, inhibiting background information reduces the cognitive load, freeing up resources for a more accurate interpretation of gaze direction. This ability is crucial for understanding where another individual is focusing and what they might be thinking or planning, thus aiding effective social communication (Baron-Cohen, 1995). By inhibiting peripheral background details, individuals can more effectively follow social cues, improving their interactions and responsiveness in social situations. This inhibition process is particularly impaired for individuals with autism, who struggle with shifting attention and interpreting the gaze for insights into others’ intentions (Dawson et al., 2004; Nation & Penny, 2008). While some research links the challenges in gaze following among individuals with autism to complex social situations (Chawarska et al., 2013; Elsabbagh et al., 2013; Nakano et al., 2010), a lack of interest in engaging with others’ mental states (Baron-Cohen, 1995), or a generic direction detection mechanism rather than a specific gaze detector (Vlamings et al., 2005), our study offers a different angle. We suggest that the difficulties in gaze following experienced by individuals with autism may be due to their inability to inhibit the background information around faces. If this hypothesis is correct, training individuals with autism to inhibit the background location could significantly improve their gaze-following capabilities.

The research area of gaze can be summarized into two aspects: identifying the function and clarifying the hardware. First, about identifying the function, since the gaze cueing effect has drawn the attention of researchers (at the end of the 20th century), it has been compared with other stimuli, such as symbolic stimulus (arrow) and peripheral stimuli. Researchers have observed the perceptual process of gaze to be faster and less susceptible to perceptual load with high automation (Driver et al., 1999; Friesen & Kingstone, 1998, 2003; Hood et al., 1998; Langton & Bruce, 1999). At first glance, these studies have demonstrated that the brain processes gaze direction differently than other directional stimuli (e.g., arrows). Essentially, these studies have shown that the processing of gaze direction occurs in a relatively independent functional module in the perceptual system. That is, the above studies have confirmed the existence of this function of gaze direction processing. Second, about clarifying the hardware, after the function of gaze was identified, the research focus has shifted to the scientific question of how our brains implement the function. Using ERP, fMRI, PET, and brain-damaging techniques, studies revealed a crucial physiological structure, i.e., the STS cortex (Akiyama et al., 2006; Shepherd, 2010). The finding of the physiological structure (or neural mechanism) of gaze direction is quite exciting, but at the same time, we think that these results do not fully answer the scientific question of how the brain realizes the function of gaze specificity. The findings on STS and gaze direction detectors suggest that our brain has a separate memory (hardware) space to calculate gaze direction, but these findings cannot discriminate what this memory (hardware) should calculate to realize the specificity of gaze direction (function). This disconnect between “hardware” and “function” is long-standing and far-reaching. On the one hand, neural structures such as STS provide the hardware basis for the specificity of gaze direction. More importantly, we argue that neural (hardware) structures may be computationally “ inhibiting background location” to exhibit specificity in the perceptual processing of gaze direction. The mechanism of inhibiting background location proposes a possible explanation of integrating behavioral and physiological evidence.

### Conclusion

In summary, when a task demand involves processing the background location, arrows produce a spatial Stroop effect but gaze does not. When a task does not involve processing the background location, both arrows and gaze produce a spatial Stroop effect. This finding suggests that inhibiting spatial background location is the reason why gaze undermine the spatial Stroop effect, which is one of the perceptual mechanisms underlying the specificity of gaze.
